# Formaldehyde Analysis in Non-Aqueous Methanol Solutions by Infrared Spectroscopy and Electrospray Ionization

**DOI:** 10.3389/fchem.2021.678112

**Published:** 2021-07-02

**Authors:** Krishna K. Barakoti, Pradeep Subedi, Farzaneh Chalyavi, Salvador Gutierrez-Portocarrero, Matthew J. Tucker, Mario A. Alpuche-Aviles

**Affiliations:** Department of Chemistry, University of Nevada, Reno, NV, United States

**Keywords:** methoxymetanol, methoxy monoglycol, methoxylated methylene glycol, ESI-TOF, hemiformal

## Abstract

We present the analysis of formaldehyde (HCHO) in anhydrous methanol (CH_3_OH) as a case study to quantify HCHO in non-aqueous samples. At higher concentrations (C > 0.07 M), we detect a product of HCHO, methoxy methanol (MM, CH_3_OCH_2_OH), by Fourier transform infrared spectroscopy, FTIR. Formaldehyde reacts with CH_3_OH, CD_3_OH, and CD_3_OD as shown by FTIR with a characteristic spectral feature around 1,195 cm^−1^ for CH_3_OH used for the qualitative detection of MM, a formaldehyde derivative in neat methanol. Ab initio calculations support this assignment. The extinction coefficient for 1,195 cm^−1^ is in the order of 1.4 × 10^2^ M^−1^cm^−1^, which makes the detection limit by FTIR in the order of 0.07 M. For lower concentrations, we performed the quantitative analysis of non-aqueous samples by derivatization with dinitrophenylhydrazine (DNPH). The derivatization uses an aqueous H_2_SO_4_ solution to yield the formaldehyde derivatized hydrazone. Ba(OH)_2_ removes sulfate ions from the derivatized samples and a final extraction with isobutyl acetate to yield a 1:1 methanol: isobutyl acetate solvent for injection for electrospray ionization (ESI). The ESI analysis gave a linear calibration curve for concentrations from 10 to 200 µM with a time-of-flight analyzer (TOF). The detection and quantification limits are 7.8 and 26 μM, respectively, for a linear correlation with *R* ^2^ > 0.99. We propose that the formaldehyde in CH_3_OH is in equilibrium with the MM species, without evidence of HCHO in solution. In the presence of water, the peaks for MM become less resolved, as expected from the well-known equilibria of HCHO that favors the formation of methylene glycol and polymeric species. Our results show that HCHO, in methanol does not exist in the aldehyde form as the main chemical species. Still, HCHO is in equilibrium between the production of MM and the formation of hydrated species in the presence of water. We demonstrate the ESI-MS analysis of HCHO from a non-aqueous TiO_2_ suspension in methanol. Detection of HCHO after illumination of the colloid indicates that methanol photooxidation yields formaldehyde in equilibrium with the solvent.

## Introduction

We present the analysis of formaldehyde in anhydrous methanol. Our motivation for this analysis stems from the need of quantifying the product of methanol photooxidation. Our aim is to use the photogenerated formaldehyde as a benchmark for the photooxidation rate in non-aqueous solvents. Here, we propose the qualitative detection of HCHO with FTIR and quantifying the generated HCHO by electrospray ionization (ESI). For the analysis, we present a derivatization method in aqueous media for the starting non-aqueous samples. Our results will show that formaldehyde is not the predominant species in MeOH because it reacts with the solvent to produce the hemiacetal methoxymethanol (MM). We propose this is a competitive equilibrium in the absence of water. It is challenging to analyze formaldehyde due to the reactivity of formaldehyde that results in coupled formaldehyde-water reactions. Formaldehyde is an important molecule to study because of its importance as a precursor in industrial chemical synthesis, research, and different applications (Walker, 1964a), such as in the synthesis of polymers, resins, and gels (Elkhatat and Al-Muhtaseb, 2011; Gaca et al., 2014; Gaca-Zajac et al., 2018). The molecule has been classified as a carcinogen by the United States National Toxicology Program (NTP National Toxicology Program, 2016) and thus, formaldehyde preservatives found in cosmetics, medications, and household products require investigation (Benassi et al., 1991; Boyer et al., 2013). It is also used as an antiseptic and one of the main ways to preserve tissues in the lab (Dubos, 1938; Maeda et al., 2014).

Our goal is to quantify the amount of formaldehyde produced under illumination at metal oxides, e.g., TiO_2_, that drives methanol’s photooxidation. While formaldehyde is the expected oxidation product, it is challenging to confirm and quantify the photooxidation product (Sun and Bolton, 1996; Wang et al., 2002). This is a similar analytical problem to detecting methanol’s electrooxidation products (Korzeniewski and Childers, 1998; Childers et al., 1999; Zhao et al., 2010; Zhao et al., 2012), where the formaldehyde yield is of mechanistic interest (Korzeniewski and Childers, 1998; Childers et al., 1999). Here, we quantify formaldehyde from the photooxidation of CH_3_OH in a non-aqueous CH_3_OH solvent. We use anhydrous methanol because we are interested in studying the photooxidation on traps near the valence band (Tamaki et al., 2006), and therefore, beyond the water’s oxidation potential. We show that the product of formaldehyde, in its aldehyde form, does not exist as the main species in either aqueous or non-aqueous solutions of CH_3_OH. Because of the broad interest in analyzing formaldehyde in consumer products, industrial and research settings, different methods are applied to measure formaldehyde (Pockard and Clark, 1984), including fluorometrically (Childers et al., 1999) from the derivative obtained with the reaction of 1,3-cyclohexadione in ammonia/ammonium acetate buffer (Dong and Dasgupta, 1987; Fan and Dasgupta, 2002). Formaldehyde in solution is challenging to analyze by IR because of the overlap with water vibration bands (Juanto et al., 1987; Korzeniewski and Childers, 1998). Similarly, in MS, the mass fragments of methanol and formaldehyde coincide, so derivatization is used in MS detection. Derivatization with 2,4 dinitrophenylhydrazine (DNPH) has been used in MS (Zhao et al., 2010; Zhao et al., 2012) adapting the procedure used to detect HCHO in gases by gas chromatography (Dalene et al., 1992).

Highly unstable in the gas form (Walker, 1964c), formaldehyde is commercially available in aqueous solutions that contain CH_3_OH. For example, a 37% aqueous formaldehyde solution is used to preserve lab tissues and is considered the most stable form of formaldehyde (Gaca-Zajac et al., 2018). Formaldehyde reacts with water to form methylene glycol and, ultimately, a long-chain polymer known as paraformaldehyde in the absence of methanol. This hydration has been known for some time and Walker reviewed it in 1964 (Walker, 1964b); more recently, the hydration products have been studied by NMR (Moedritzer and Wazer, 1966; Dankelman and Daemen, 1976; Hahnenstein et al., 1994; Gaca et al., 2014). In water at room temperature, the equilibrium favors the formation of methylene glycol, Eq. 1, and the equilibrium constant has been reported, *K*_h_ = 1.3 ×10^3^ at room temperature (Winkelman et al., 2002).

$$\text{HCHO }+{\text{ H}}_{2}\text{O}⇌\text{ HO}-\text{C}{\text{H}}_{2}-\text{OH}$$ (1)

Methanol is added to aqueous formaldehyde solutions to stabilize the mixture by stopping polymerization. Previous reports (Gaca et al., 2014; Gaca-Zajac et al., 2018) indicate that formaldehyde forms methoxylated methylene glycol or methoxymethanol, CH_3_O-CH_2_-OH, according to Eq. 2.

$$\text{HO}-\text{C}{\text{H}}_{2}-\text{OH }+{\text{ CH}}_{3}\text{OH }⇌{\text{ CH}}_{3}\text{O}-\text{C}{\text{H}}_{2}-\text{OH }+{\text{ H}}_{2}\text{O}$$ (2)

However, these experiments were performed in methanol containing water, e.g., the reports of Gaca et al. (Gaca et al., 2014; Gaca-Zajac et al., 2018) The authors studied (Gaca et al., 2014) solutions with a formaldehyde mole fraction of 0.063 to 0.006, a methanol mole fraction of 0.02–0.07, and a water mole fraction from 0.1 to 0.3. Also, in many reports, the starting reactant is an aqueous solution of HCHO, thus introducing hydrated species. Formaldehyde also forms diglycol, triglycol, and other products following the initial hydration, and ultimately, larger polymeric species (Gaca-Zajac et al., 2018). In an experiment performed with methanol from a commercial source (ACS grade) and used as received and an aqueous formaldehyde standard solution, Gaca et al. (2014) found that the equilibrium favors methylene glycol and its polymeric form in excess water. In contrast, it favors methoxymethanol in excess methanol (Gaca-Zajac et al., 2018).

We are interested in the methanol-formaldehyde equilibrium because of its implications in quantifying the methanol photooxidation in photocatalytic reactions. Light absorption by a semiconductor particle, like TiO_2_ generates a valence band hole. This process drives methanol oxidation to formaldehyde via hydroxy radicals in aqueous solutions (Sun and Bolton, 1996; Wang et al., 2002), with the OH^•^ generation rate recently quantified (Zigah et al., 2012). We have used anhydrous, neat CH_3_OH as a case study for the photocatalytic activity (Fernando et al., 2013; Fernando et al., 2016) of semiconductor nanoparticles (NPs) which models of non-aqueous solvents without the complications of water oxidation and pH effects and interesting for the reactivity of trapped holes (Tamaki et al., 2006). However, we are not aware of methods to detect formaldehyde in anhydrous methanol, and here, we present the qualitative and quantitative detection of HCHO derivatives. We present evidence of the reaction of HCHO with anhydrous methanol that yields MM at room temperature, reaction (Eq. 3):

$$\text{HCHO}+{\text{CH}}_{3}\text{OH}⇌\text{ OH}-\text{C}{\text{H}}_{2}-\text{O}-\text{C}{\text{H}}_{3}$$ (3)

Although it is known that, in general, aldehydes react with alcohols to form hemiacetals (Ashdown and Kletz, 1948) and hemiketals, the spectra of the product of formaldehyde and methanol has not been documented. There is no conclusive spectroscopic evidence of the hemiacetal formation from anhydrous MeOH mixed with HCHO in the liquid phase to the best of our knowledge. For example, in 1898, Delépine (1898) reported that a concentrated solution of formaldehyde in CH_3_OH boiled at 96°C, over 30°C above the normal boiling point of the solvent (64.7°C). In 1933, Walker (1933) reported that liquid formaldehyde at −80°C mixes with MeOH but later reacts to form a solid; after heating the product, a clear solution was obtained. However, we are not aware of the isolation of MM or its spectroscopic characterization, possibly because, in more recent reports, the precursors are aqueous solutions of HCHO, where HCHO hydration, reaction (Eq. 1) has already occurred. Formation of MM is thought to occur after hydration, as in reaction (Eq. 2). Peaks of FTIR, Raman, and NMR spectroscopies have been assigned to methoxymethanol in mixtures that contain water, CH_3_OH, and HCHO (Gaca et al., 2014; Gaca-Zajac et al., 2018). Also, MM and other byproducts were detected during the photolysis of methanol studied as a function of pressure from 1 bar to 1.8 GPa (Fanetti et al., 2011). Johnson and Stanley irradiated MeOH with an IR laser to make formaldehyde in excess methanol, and they assigned some of the IR peaks to methoxymethanol (Johnson and Stanley, 1991). The authors obtained the MM spectra from a gas chromatography column at low temperatures (−16 to −60°C) and reported MM to be unstable at higher temperatures (Johnson and Stanley, 1991). Methoxymethanol has been reported to form when formaldehyde is bubbled through methanol solution, although water was added to these mixtures (Hahnenstein et al., 1994; Celik et al., 2008). Vibrational bands of water overlap with those of MM, formaldehyde, and other small molecules, and this complicates FTIR analysis of HCHO in aqueous media (Dong and Dasgupta, 1986). An NMR study considered hemiacetals formation in a HCHO, CH_3_OH, and water (or D_2_O) mixture, for HO(CH_2_O)_n_CH_3_ but only for *n* > 1, thus excluding the possibility of methoxymethanol (Hahnenstein et al., 1994).

In this work, we report the FTIR spectra of formaldehyde in various non-aqueous methanol solutions, and we show that methoxymethanol is the main product. We also study the differences between deuterated (CD_3_-OH and CD_3_-OD) and non-deuterated methanol solutions. To the best of our knowledge, this is the first report of formaldehyde IR spectra in completely deuterated methanol, CD_3_OD. We show that the vibrational bands obtained with FTIR for these solvents correspond to the reaction of HCHO with methanol and the formation of MM. Also, we describe a method for quantitative analysis of formaldehyde in anhydrous methanol by modifying a technique for aqueous detection by ESI-TOF MS. Our approach is based on the derivatization to formaldehyde-2, 4-dinitrophenylhydrazone from 2, 4 dinitrophenyl hydrazine (DNPH). The derivatization occurs in an aqueous acid media, and we modified the conditions for the detection by ESI of HCHO in non-aqueous samples. We present a benchtop method adapted from the ESI analysis from aqueous samples in an online setup (Zhao et al., 2010; Zhao et al., 2012). The first report using DNPH to detect HCHO was published by Fracchia et al. (1967) to collect the derivatized product from gaseous samples in an aqueous trap. The products of the reaction with DNPH were used to determine the components of aldehydes in a mixture by GC. Fung and Grosjean optimized the derivatization of HCHO to use it with high pressure liquid chromatography (HPLC); the optimized method allowed detection of nanograms of HCHO in the injection loop out of a mixture of aldehydes (Fung and Grosjean, 1981). Lowe et al. discussed experimental issues with the derivatization conditions and suggested best practices due to stability concerns of the derivatized hydrazone (Lowe et al., 1981). In this report, we use the derivatization reaction (Fracchia et al., 1967; Papa and Turner, 1972; Fung and Grosjean, 1981; Lowe et al., 1981) in non-aqueous methanol for analysis ESI-TOF MS. Key to enable the analysis of ESI-TOF in this non-aqueous solvent is the sample preparation steps that include removal of sulfates and a liquid extraction in a solvent compatible with ESI.

## Experimental

### Materials and Reagents

Titanium (IV) isopropoxide (97%), glacial acetic acid (≥99.8%), 2-propanol (≥99.8%) and spectrophotometric grade methanol (≥99.9%), formaldehyde solution (37.5% w/w), 2, 4 dinitrophenyl hydrazine, formaldehyde 2, 4-Dinitrophenyl hydrazine, paraformaldehyde, deuterated methanol-d3 (99.8 atom % D) and -d4 (99.8 atom % D) were obtained from Sigma Aldrich chemicals. Sulfuric acid (ACS reagent grade) was purchased from Pharmaco-Aaper. Methanol was dried with activated alumina at least a week inside an Ar glove box with partial pressures for water and oxygen P(H_2_O) and P(O_2_) < 0.1 ppm. All other chemicals were used as received. For the aqueous solutions, we used water of 18 MΩ cm from a purification system (Barnstead).

### Anhydrous Formaldehyde Sample Preparation

Paraformaldehyde was cracked to gaseous formaldehyde and captured in dried methanol. In the Supplementary Material (SM), we present a schematic of the experimental setup (Supplementary Figure S1). Two custom glass tubes sealed to make a flat surface with a thread on the other end were taken for the cracking experiment connected with PTFE tubing. The tubing was fixed with two rubber septa to make the connection airtight. One of the tubes was filled with 1 ml of anhydrous methanol, and the other tube was filled with 1 g of paraformaldehyde. The apparatus was assembled inside the Ar glove box and moved outside after sealing it airtight. The paraformaldehyde cracking was performed outside the glove box and inside a laboratory hood. Paraformaldehyde powder (Sigma Aldrich, MO, United States) was heated to 110°C, which we monitored with an IR thermometer after calibration to a reading of 210°C (average reading of glass container and the paraformaldehyde heating up while the heating plate was around 260°C). The released formaldehyde was captured in methanol by bubbling the gas through the solvent.

### FTIR Experiment

A specially designed infrared cell was used for the measurements. Supplementary Figure S2 depicts the cell in the SI. The cell was machined in-house to provide a small pathlength, b < 1 mm, for two partitions with a calcium fluoride (CaF_2_) window. The CaF_2_ glass sandwiched a polytetrafluoroethylene spacer (100 μm, unless otherwise noted) to define the pathlength while making two unconnected compartments. The spacer was held by a rubber gasket and a stainless-steel cell frame and heated on a plate for 5 min at 150°C for tight sealing. After the cell cooled to room temperature, the cell was transferred to the Ar glovebox to fill both compartments with 20 µl of solutions using a Hamilton syringe. One of the compartments had the sample solution, while the other contained a blank (anhydrous MeOH or the deuterated species). After loading the sample and blank, a rubber septum and metal screw sealed the injection ports before taking the cell outside the glovebox. Aliquots of the final sample were dried in the glove box to determine the mass of paraformaldehyde precipitated after allowing the MeOH to evaporate.

We acquired IR spectra on an FTIR spectrometer (ThermoNicolet 6700), purged with nitrogen gas. The detector was a liquid nitrogen-cooled mercury cadmium telluride (MCT) transducer. The cell and detector were customized in an external benchtop using a setup previously described (Waegele et al., 2009; Williams et al., 2011). Briefly, a stepper motor switched the cell compartments to alternately expose the analyte and blank compartments to the beam path. This setup collected FTIR spectra under nearly identical experimental conditions in single beam mode with a resolution of 1 cm^−1^. Typically, 100 scans were averaged per spectrum, and spectra showed here are blank corrected unless noted otherwise. For example, the blank measurements used a cell with neat methanol in one compartment to make it optically equivalent to the HCHO/MeOH mixture.

To study the effect of water on CH_3_OH-HCHO, the required amount of water to prepare different aqueous solutions was added from a CH_3_OH water solution in the glovebox. Pure anhydrous methanol was used as a background for every FTIR measurement and was subtracted from the formaldehyde in methanol spectra to get the spectra of formaldehyde.

### Gaussian09 Computations

An anharmonic frequency calculation of methoxy methanol was performed using a B3LYP/6-31+G* level of theory. The computation was used as a guide to assign vibrational transitions to normal modes.

### ESI Measurements

We derivatized the samples on the benchtop and prepared them for ESI-TOF analysis of formaldehyde in anhydrous methanol solutions. The derivatization of formaldehyde by 2, 4-DNPH forms formaldehyde 2, 4-Dinitropheyl hydrazone (FDH), an easily ionizable species (Zhao et al., 2010). This scheme is based on the seminal report by Fracchia et al. (1967) who first demonstrated this reaction for analytical applications. Zhao et al. (2010) recently demonstrated ESI detection of the derivatized FDH to study the electrocatalytic products of methanol oxidation in an aqueous phase. These authors used an online, continuous microfluidic setup to inject the FDH product in an organic phase to facilitate detection from an aqueous sample.

**FIGURE 1:**
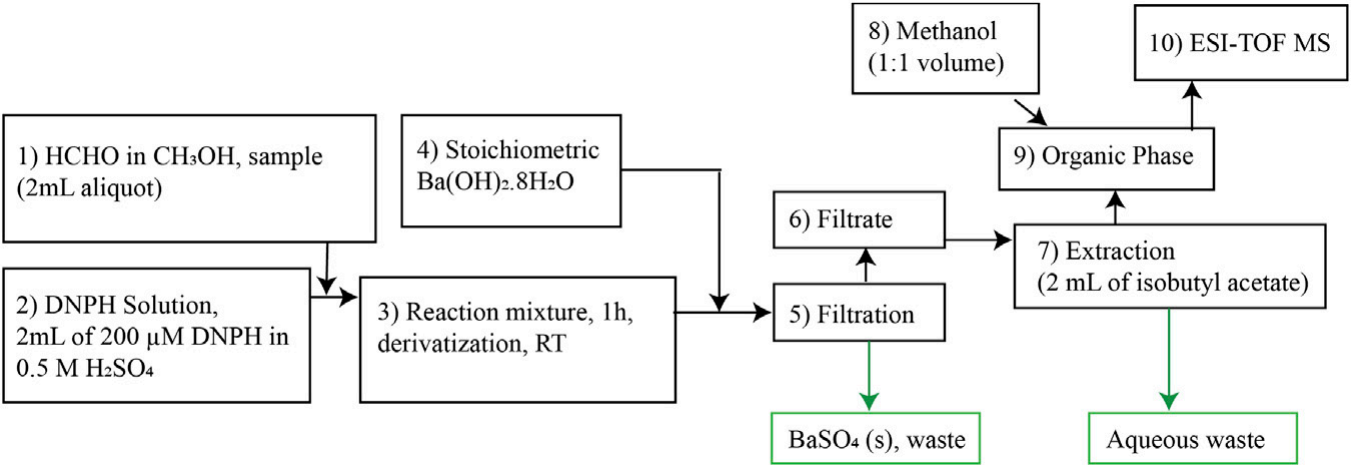
Flow diagram for formaldehyde derivatization for ESI-TOF MS analysis.

We adapted the procedure from ref (Zhao et al., 2010) 1) to enable us to perform the analytical manipulation on the benchtop without the inline derivatization setup. 2) To allow us to extract the derivatized product, FDH, from the anhydrous organic phase. Figure 1 is a schematic representation of the optimized procedure; we include a discussion of how we developed the protocol below with the following steps 1) The formaldehyde derivatization reaction was performed by mixing the formaldehyde in methanol solution in the 1–100 μM range. 2) A 2-ml sample aliquot was mixed with 200 µM of 2, 4-DNPH made in 0.5 M sulfuric acid (2 ml) solution at room temperature. 3) The derivatization reaction was carried out for 1 h 4) Barium hydroxide octahydrate, Ba(OH)_2_•8H_2_O, was added to the reaction mixture in a stoichiometric amount. Barium hydroxide neutralizes the sulfuric acid and produces barium sulfate. 5) The barium sulfate was separated out from the reaction mixture using a 0.2 µm Whatman PTFE syringe filter. 6) Then, the analyte present in the filtrate (2 ml) was 7) extracted with 2 ml of isobutyl acetate (organic phase). In steps 8 and 9, The analyte-containing isobutyl acetate, obtained by extraction, was mixed with methanol in a 1:1 ratio by volume. 10) The final mixture of methanol and isobutyl acetate containing the formaldehyde derivative was delivered to a high-resolution mass spectrometer. An Agilent Technologies, G6230B TOF-LC/MS was used for the measurements. The ion source used for the measurements was electrospray ionization with a time of flight (TOF) analyzer. Typical injection settings were syringe: flow rate of 1,000 µl/h for ionization. The fragmentor and skimmer voltage used for the measurement were 175 and 65 V, respectively and the gas temperature was 325°C. We produced the parent ion from formaldehyde 2, 4-dinitrophenyl hydrazone (m/z = 209.01), following ionization in the negative ion mode. We validated the signals with FDH and 2, 4-DNPH standards (Sigma Aldrich). The FDH standard was stored in an Ar glovebox.

We obtained a calibration curve from standard solutions of formaldehyde from 10 to 200 µM. The standards were prepared by dissolving a HCHO commercial standard (37% aqueous solution, Sigma) in neat MeOH under ambient conditions. Formaldehyde solutions were derivatized with 2, 4-DNPH, and analyzed by ESI-TOF-MS. The signal intensities of the mass fragment (m/z = 209.01) were corrected for the method blank, prepared by taking neat methanol through the derivatization steps (Figure 1) and measuring the signal intensities at m/z = 209.01.

## Results and Discussion

### FTIR

**FIGURE 2:**
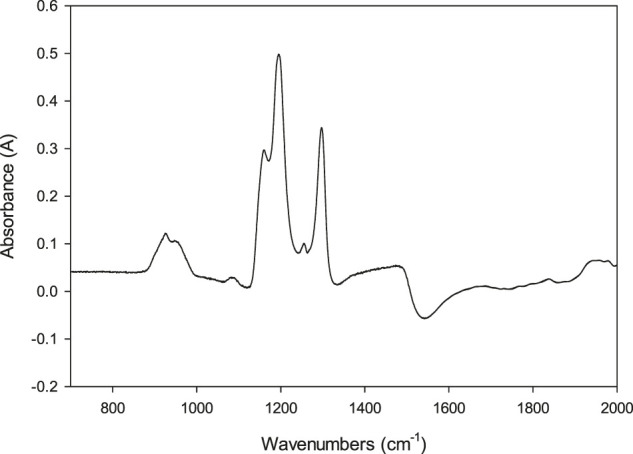
FTIR spectrum of methoxymethanol prepared by dissolving formaldehyde in anhydrous methanol CH_3_OH. Pathlength 153 μm determined by interferometry.

Figure 2 shows the subtracted FTIR spectrum of formaldehyde in anhydrous methanol (CH_3_OH) in the 700–2,000 cm^−1^ region, which is the region of interest for the MM characterization. In the SM, we present the single beam spectra for the CH_3_OH and the MM spectra obtained by bubbling HCHO into CH_3_OH. Supplementary Figure S3A shows the full spectrum, and Supplementary Figure S3B the detail in the 800–2000 cm^−1^ region. Note that above 2,000 cm^−1^ the spectra overlap between the blank and sample is large, so we do not discuss this region here. Therefore, we study the region characteristic of methoxy methanol because of its relevance in qualitative analysis. The spectrum in Figure 2 is consistent with the formation of MM because: 1) the characteristic peak for the carbonyl group is not present around 1,700–1,800 cm^−1^. Note that spectra in Supplementary Figure S3A,B, the detail in the 800 to 2,000 region show that the detector is not saturated in the 1,700–1,800 cm^−1^ region, indicating that the absence of a carbonyl peak is not a problem of the background subtraction. 2) There are strong absorption peaks at 930, 1,116, 1,195, and 1,297 cm^−1^. These vibrational peaks and the lack of characteristic peaks for HCHO are associated with a hemiacetal (Ashdown and Kletz, 1948). Here, we assign the peaks in the 900 to 1,500 cm^−1^, shown in Figure 2, to methoxymethanol formed from formaldehyde and CH_3_OH, in excess anhydrous CH_3_OH (solvent). We tentatively assign the modes based on previous reports. The band at 930 cm^−1^ is close to peaks assigned by Gaca et al. (Gaca-Zajac et al., 2018) and Wrobel et al. to the symmetric stretch COC (Wrobel et al., 1999) However, Gaca et al. (2018) used aqueous precursors, which could account for the difference in peak position, while Wrobel used an Ar matrix at 10 K. In our spectra, we observed three closely spaced peaks at 1,116, 1,195, and 1,297 cm^−1^ that has not been reported before. A Gaussian 09 DFT frequency calculation using a B3PW91/6-31++G (d,p) level of theory for methoxy methanol predicts three transitions in this region of the spectrum. The normal modes for each of these transitions involve the COC moiety motions are highlighted in the SM information (Supplementary Figure S5). Wrobel et al. assigned the strongest peak in their spectra, 1,125 cm^−1^ to the stretch of COC and the ω (torsion) mode of CH_2_. Interestingly, Gaca et al. did not observe this peak in their experiments in aqueous CH_3_OH solutions and assigned their strongest peak at 1,025 cm^−1^ to methylene glycol (Gaca-Zajac et al., 2018), the product of HCHO and H_2_O, Eq. 1. We propose that the absence of the peak at 1,025 cm^−1^, previously assigned to MM, is consistent with our procedure to minimize water in the solutions. Therefore, we assign the bands at 1,116, 1,195, and 1,297 cm^−1^ to the COC bond between CH_3_-O-CH_2_ in MM. Our observations are consistent with the above ab initio calculations and Johnson and Stanley’s findings (Johnson and Stanley, 1991) who also observed characteristic peaks between 930 and 1,450 cm in the gas phase. They obtained spectra in a column at 6°C and after the products of CD_3_OH photolysis were separated in a gas chromatography column. The spectra were assigned to MM, but the authors did not assign the peaks to vibrational modes. Also, Faneti et al. (2011) irradiated CH_3_OH and listed peaks without mode assignments that the authors assigned to MM. It is also worth noting that the contributions of different isomers of MM are expected to change widely under different matrices and temperatures, as discussed before (Fanetti et al., 2011; Gaca-Zajac et al., 2018).

**FIGURE 3:**
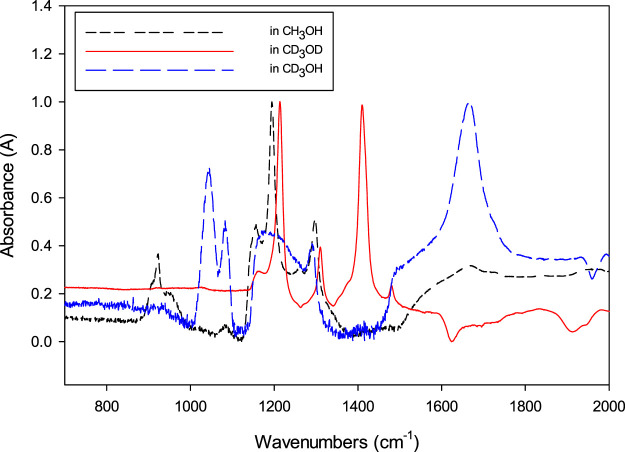
Effect of deuterium on the FTIR spectrum of MM. Methoxymethanol prepared by dissolving formaldehyde in (−− −, black) anhydrous CH_3_OH, in (⋅⋅⋅⋅, red) CD_3_OD, and in (---, blue) in CD_3_OH. The spectra were normalized to the strongest peak and corrected by the background of CH3OH, CD_3_OH, and CD_3_OD.

Figure 3 shows the effect of deuterated methanol in the spectra of the MM produced by bubbling HCHO into the deuterated methanol samples. Interestingly, the peak at 925 cm^−1^ assigned to the stretching of COC shifts to lower frequencies and decreases in intensity, consistent with the findings ofFanetti et al. (2011), where they irradiated CH_3_OH and CD_3_OH and observed the peak shift to lower wavenumbers. The peaks related to the COC bond shift towards higher wavenumber in the order of CH_3_OH < CD_3_OH < CD_3_OD, which could be due to different solvachromatic effects on the differently substituted compounds, both direct and indirect contributions. An alternative explanation is that resonant frequency combination or overtone bands may couple to the fundamental mode due to Fermi resonances or wavefunction mixing, leading to shifting of the dominant mode. We are currently investigating these possibilities, but the isotopic effect confirms that the peaks are related to the formation of MM: CH_3_O-CH_2_-OH and its deuterated analogs.

The full spectra assignment and other spectroscopic properties of MM in the three solvents are beyond the scope of this paper, and they will be reported elsewhere. Here, we present the effect of deuteration as evidence that the peaks around the 1,195 cm^−1^ region are due to the formation of MM from HCHO reacting with methanol. Thus, we propose to use these peaks for the qualitative analysis of MM, specifically the peaks around 1,195 cm^−1^ in CH_3_OH. Interestingly, these peaks overlap with the broad absorption band of MeOH around 1,100 cm^−1^, which is usually assigned to C-O stretching in alcohols (Socrates, 2001). The spectra obtained from the solution and the CH_3_OH background are shown in the SI, Supplementary Figure S3. Supplementary Figure S3A shows the full spectra collected, which, as expected, shows that many of the peaks of MM and CH_3_OH overlap. Interestingly, we can detect the peaks that overlap with the methanol C-O stretching mode because the CH_3_OH absorption decreases and has a valley around 1,250 cm^−1^, the region where the MM characteristic peaks appear (cf. Supplementary Figure S3B). We estimated the sample concentration for the spectra shown in Figure 2 to be 0.35 M, based on the evaporation of the solvent and measuring the weight of the paraformaldehyde that precipitated. This makes the extinction coefficient for 1,195 cm^−1^, with A = 0.50, ε = 1.47 × 10^2^ M^−1^ cm^−1^. These results are consistent with the 1,195 vibration for MM having a stronger extinction coefficient than CH_3_OH: for neat CH_3_OH, at 1,195 cm^−1^ with C = 24.7 M and A = 1.32 (Supplementary Figure S3B), then ε = 5._3_ M^−1^ cm^−1^. That is, MM has a larger absorption cross-section than CH_3_OH. The large spectral overlap shown in Supplementary Figure S3 makes it difficult to quantify MM with our current setup, and in turn, determine the HCHO concentration with FTIR. We can measure absorbances of around 0.1 above the larger background of A = 1.3 for the CH_3_OH solvent, which corresponds to a HCHO equivalent concentration of ca. 70 mM.

### Effect of Water

A sample was prepared by adding a large excess of water: 150 μl of water was added to 1 ml formaldehyde methanol solution prepared by cracking paraformaldehyde as described above. Supplementary Figure S4 shows the spectra of formaldehyde in methanol where bands at 1,670 and 3,600 cm^−1^ appear while the peaks at 1,116, 1,195, and 1,297 cm^−1^ decrease in intensity and form a broader absorption envelope. This spectrum change is consistent with hydrates species shifting the equilibrium from methoxymethanol to methylene glycol (OH-CH_2_-OH) because the equilibrium constant for HCHO hydration, *K*_h_ = 1.3 × 10^3^ favors methylene glycol (Winkelman et al., 2002). Further, a strong adsorption band at 1,670 cm^−1^ is consistent with water in the methanol solution, along with the strong absorption band at 3,600 cm^−1^ due to water’s O-H stretching.

### Quantifying Formaldehyde by ESI

**FIGURE 4:**
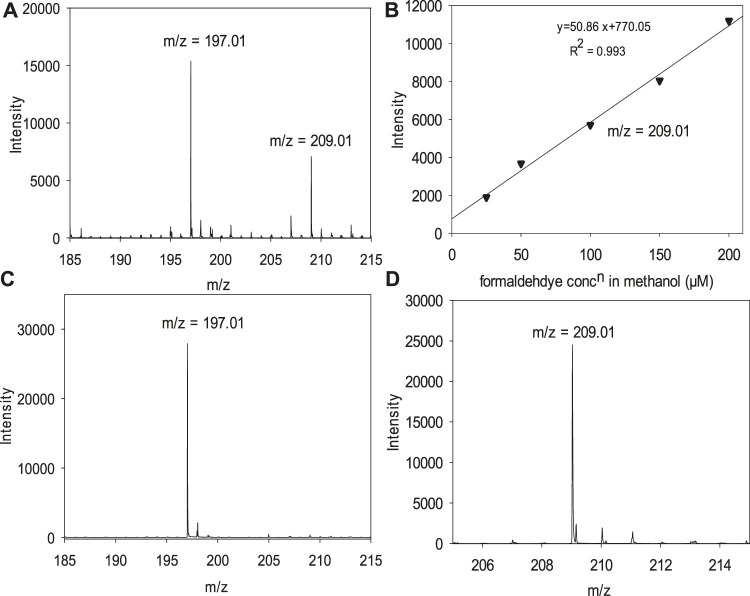
ESI spectra and calibration curve for formaldehyde in methanol after derivatization to formaldehyde 2, 4-Dinitrophenyl hydrazone. **(A)** Spectrum for a derivatized 150 µM formaldehyde sample in MeOH containing DNPH, m/z = 197 and FDH, m/z = 209.01. **(B)** Calibration curve obtained for formaldehyde in methanol after derivatization plotting the intensity of the parent ion of FDH, m/z = 209. **(C)** Spectrum for 2, 4-DNPH (standard). **(D)** Spectrum for FDH (standard).

**FIGURE 5:**
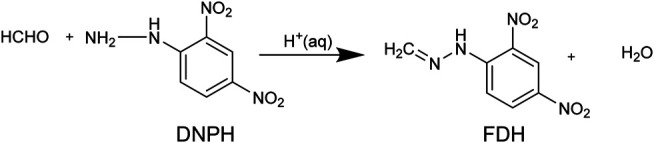
Formaldehyde derivatization reaction used in this work. We modified the method to enable analysis from non-aqueous methanol because the aqueous acid solution complicates the separation of the formaldehyde 2, 4-dinitrophenyl hydrazone (FDH).

We use an ESI-MS detection method for 10–200 μM of HCHO. It was necessary to modify the method reported byZhao et al. (2010) for detecting HCHO in anhydrous MeOH. We chose this method because Zhao et al. demonstrated that the detection of HCHO in MeOH/water mixtures and that formic acid is not a strong interferent. We modified the protocol to derivatize HCHO on the benchtop to use FDH for analysis. As we discuss below, simple extraction from the non-aqueous matrix did not yield satisfactory results. In our optimized method, the analytical signal is the FDH parent ion’s intensity in the negative ion mode, which is facilitated by FDH that readily loses a proton (Zhao et al., 2010). Figure 4 shows the calibration curve of the modified method and some validation experiments. Standard formaldehyde solutions in different concentrations from 10 to 200 µM were prepared in aq. 0.5 M sulfuric acid solution and were derivatized with 200 µM DNPH in aq. 0.5 M sulfuric acid solution (Figure 1). Figure 4A is the mass spectrum of a derivatized HCHO sample after organic extraction, where the mass fragments of 197.01 and 209.01 are assigned to DNPH and FDH. Figure 4B is the calibration curve using the m/z = 209.01 corresponding to the FDH parent ion. We validated the signals with DNPH and FDH standards (Sigma Aldrich), with the MS spectra shown in Figure 4C,D, respectively. These standards were analyzed in CH_3_OH with a 1:1 v/v isobutyl acetate mixture. The FDH standard yielded calibration curves consistent with the curves obtained following the modified formaldehyde derivatization protocol in Figure 4B. The derivatization reaction described above was performed for 1 h. After that, we found that it was key to treat the derivative product with Ba(OH)_2,_ and the derivatization product was extracted with isobutyl acetate. The analyte mixture extracted in isobutyl acetate was mixed with methanol in a 1:1 (v/v) ratio before their measurements using ESI-TOF mass spectrometer. Based on these standards, the extraction step after the derivatization yields an isobutyl mixture containing residual DNPH, initially added in excess, and the HCHO derivatization product (Figure 5). We determined residual sulfate to be detrimental for the analysis and preventing analysis of HCHO in anhydrous CH_3_OH. The extraction, as reported by Zhao (Zhao et al., 2010; Zhao et al., 2012), was not effective because the non-aqueous solvent remains in the derivatization mixture. The non-aqueous solvent is miscible in water and isobutyl acetate, and this causes the sulfate ion to partition into the organic phase in the extraction with isobutyl acetate. Our modified protocol effectively removed sulfuric acid in the aqueous reaction mixture used to derivatize the analyte and yields a final isobutyl acetate organic phase with a negligible bisulfate or acid content. We propose that this would be a common feature to other analyses in non-aqueous solvents.

### Method Optimization

Our initial attempt to quantify HCHO without removing sulfate in anhydrous MeOH resulted in a calibration curve with lower intensity and a relatively low *R* ^2^ of 0.94 (SI, Supplementary Figure S6). We assign this deviation from linear behavior to traces of sulfate ions present in the organic phase, as shown in the SI. Supplementary Figure S7 shows the spectra that we assign to bisulfate, m/z = 96.98, and the effect of bisulfate is consistent with Zhao's report (Zhao et al., 2010). Because isobutyl acetate and methanol are miscible solvents, sulfuric acid mixed in the methanol during the derivatization reaction (step [3] in Figure 1) ends in the organic phase after extraction (step [7] in Figure 1). Also, the formaldehyde solution obtained from Sigma Aldrich chemicals used in this measurement contains 12% v/v of methanol as a stabilizer. Here, we introduce a new approach to neutralize the sulfuric acid and remove HSO_4_ (present in the extracted analyte mixture in isobutyl acetate, step [4].

We attempted to use a rotary evaporator and vacuum techniques to remove sulfuric acid from the solution mixture. However, heating the mixture caused the analyte to decompose because the derivatized product, FDH, is thermally unstable (Papa and Turner, 1972). Therefore, we decided to remove sulfates from the derivatization mixture by neutralization with Ba(OH)_2_, step [3]. We tested several bases for the neutralization, but because salts of sodium and potassium sulfate are soluble in reaction mixtures and cannot be easily removed, we considered sodium hydroxide and potassium hydroxide not advisable. In our first attempt to remove sulfate ions, we introduced ammonium hydroxide to the extracted organic phase. Ammonium hydroxide reacts with sulfuric acid readily and forms ammonium sulfate. The ammonium sulfate produced from the neutralization is volatile, and in low concentration, we expected to be tolerable to ESI, with minimal interferences. However, the results we obtained for different solutions after NH_4_OH treatment while increasing the analytical response (ion counts) did not provide a linear calibration curve (Supplementary Figure S8). We also tested barium carbonate to neutralize the sulfuric acid in the reaction mixture. We observed that reaction with barium carbonate (BaCO_3_) was not successful in the methanol-water solvent because the pH of the reaction mixture (step [3]) did not increase above 1.83 even after stirring 48 h at 40 C on a hot plate.

We introduced barium hydroxide octahydrate, Ba(OH)_2_∙8H_2_O as a suitable reagent to achieve our goal, step [4] in our optimized procedure. Barium hydroxide reacts with sulfuric acid and produces a barium sulfate, a white insoluble precipitate with *K*_sp_ = 1.08 × 10^–10^ [ref CRC and Lide (2004)], separated by the filtration using 0.2 µm pore size Whatman filter. This reaction and filtration eliminate the bulk sulfate constituents from the solution and protect the ESI components from corrosion. However, in our initial attempts to use the filtrate directly for the ESI analysis, we did not reproducibly achieve neutral filtrate solutions from the different concentrations of analyte prepared. This poor reproducibility indicates the traces of sulfuric acid or barium hydroxide remain in the aqueous filtrate as unreacted reagents. As discussed earlier, sulfuric acid suppressed the ion counts in the ESI, and the calibration curve for the derivative product FDH deviates from linearity. Therefore, we further extracted the analyte by isobutyl acetate to remove the traces of unreacted acid and basic reagents (steps [7–9]). A control of FDH in an isobutyl acetate: methanol mixture gave satisfactory mass spectra (Supplementary Figure S9A) and a calibration curve with R^2^ > 0.99 (Supplementary Figure S9B).

To test the optimized procedure, we prepared the standard formaldehyde solutions in different concentrations from 10 to 200 µM in methanol solution and were derivatized with 2, 4-DNPH solution prepared in 0.5 M H_2_SO_4_ solution. We quantified formaldehyde from the signal intensities of mass fragments, m/z = 209, for the devitalized formaldehyde 2, 4-Dinitrophenyl hydrazone. We obtained a calibration curve with a limit of detection (LOD) and quantification (LOQ) for formaldehyde of 7.75 and 25.85 µM, respectively, taking the 3 *s*/*m* and 10 *s*/*m* criteria. The calibration curve is linear with R^2^ > 0.99 over the concentration range of formaldehyde from 10 to 200 µM made in methanol. The calibration curve obtained in the measurements is shown in Figure 4B. The results herein demonstrated the validity of the proposed approach and underpinned the method's suitability for quantitative measurements of formaldehyde in methanol solution, i.e., in non-aqueous samples. This is the first report for quantitative measurements of formaldehyde at micro molar concentrations in a non-aqueous solvent to the best of our knowledge.

**FIGURE 6:**
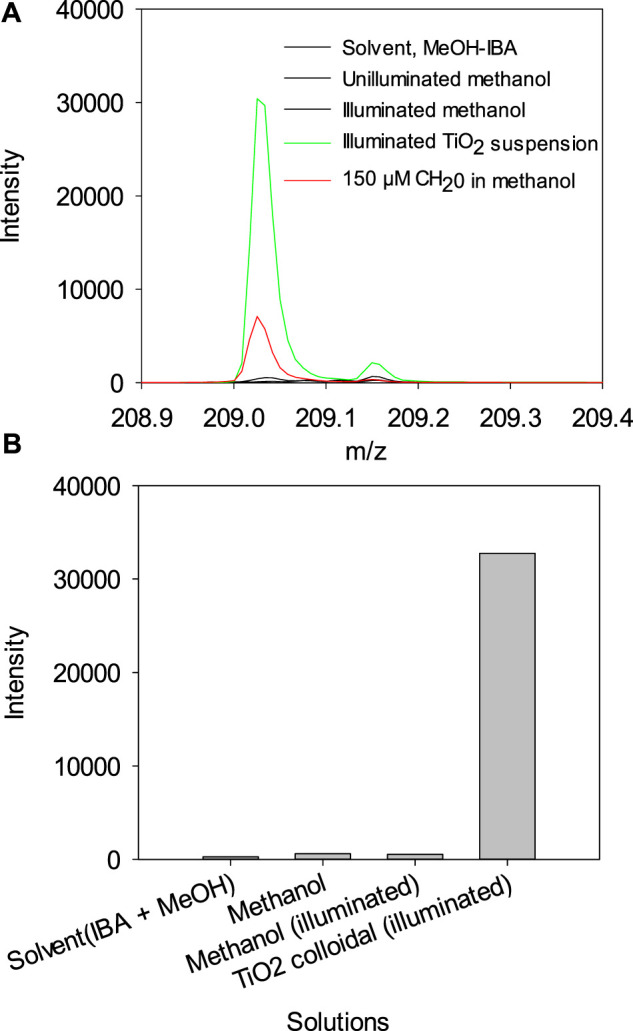
**(A)** Mass spectra for the formaldehyde 2, 4-Dinitrophenyl hydrazone region obtained for different solutions. **(B)** Histogram showing the abundance of formaldehyde 2, 4-Dinitrophenyl hydrazone (m/z = 209) obtained for different solutions.

We show that illumination of a TiO_2_ suspension in CH_3_OH yields HCHO. A suspension of TiO_2_ was illuminated for 15 h with a 150 W Xe Arc lamp. The suspension was treated with the same method as the HCHO standard, and the results are shown in Figure 6A. The mass spectra for the derivatized product, m/z = 209, show the reaction's product if HCHO in neat methanol. Note that illuminated methanol control has significantly lower HCHO (Figure 6A,B), indicating that HCHO is formed under photocatalytic conditions, irradiated with a broad spectrum source.

**FIGURE 7:**
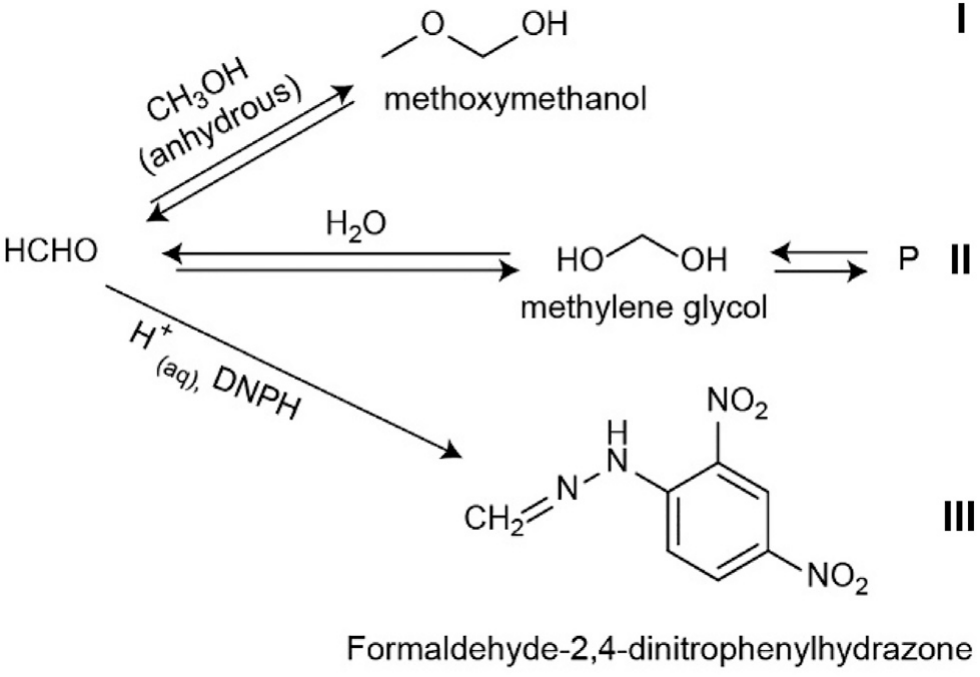
Proposed equilibria for the analysis of formaldehyde. In anhydrous methanol solutions, we demonstrate the formation of MM by FTIR reaction I) and can be used to prove the presence of HCHO qualitatively. In aqueous solutions, MM formation competes with the formation of methylene glycol and subsequent species II) and the derivatization reaction III). In acid aqueous media reaction III) ultimately yields the derivatized FDH used for ESI analysis.

A final note about the analysis of HCHO from non-aqueous CH_3_OH from FTIR and after derivatization is on the implications for HCHO analysis, as depicted in Figure 7. As described above, the reaction of HCHO with CH_3_OH yields MM. This compound has been observed in solutions that included water, and therefore hydrated species. Methoxymethanal was prepared from aqueous precursors (Gaca et al., 2014; Gaca-Zajac et al., 2018) and irradiating CH_3_OH used as received (Johnson and Stanley, 1991; Fanetti et al., 2011) and thus, containing water. However, these prior results are consistent with our MM detection, with the main difference that in this work, the starting reactant was CH_3_OH dried thoroughly, which corresponds to reaction (*i*) in Figure 7. In the anhydrous methanol solution, the FTIR spectra show no evidence of a peak that corresponds to the double bond H_2_C=O, as discussed above, which indicates that in neat CH_3_OH, the main chemical species is the hemiacetal MM. Reaction (*ii*) in Figure 7 is the well-known hydration of HCHO that ultimately yields oligomers. Because derivatization of anhydrous samples with aqueous DNPH yields the resulting FDH from the aldehyde derivatization, it follows that HCHO must be in equilibrium with MM. As reaction (*iii*) proceeds, the equilibrium in reactions (*i*) and (*ii*) must shift to produce HCHO, which in turn yields the derivatized hydrazone. We point out that these structures are consistent with 2D NMR studies for both methoxymethanol and FDH, as the structures in solution. However, a full discussion of the NMR results, and the effect of TFA functionalization (Crespi et al., 2018) is beyond the scope of this paper and will be presented in due time (Subedi et al., manuscript in preparation)^1^.

## Conclusion

We have demonstrated the formaldehyde analysis in CH_3_OH as a case study to detect HCHO in non-aqueous samples. At higher concentrations, we detect the product of HCHO with CH_3_OH to be methoxy methanol (MM, CH_3_OCH_2_OH) by FTIR. The spectral feature around 1,195 cm^−1^ can be used to qualitatively detect formaldehyde after reacting it with neat CH_3_OH. We estimate our current limit of detection to be 70 mM for the FTIR setup. To quantify HCHO in CH_3_OH, we demonstrated the derivatization with DNPH in an aqueous H_2_SO_4_ solution. We measure the derivatized FDH, in concentrations from 10 to 200 µM with the optimized procedure shown in Figure 1. This protocol yielded a limit of detection and quantification of 7.8 and 26 μM, respectively, for a linear calibration curve with R^2^ > 0.99. Key to the ESI analysis of HCHO is the use of Ba(OH)_2_ to remove sulfate ions from the derivatized samples, followed by extraction with isobutyl acetate. Based on the FTIR results, most of the HCHO exists in the form of MM in dry CH_3_OH. In the presence of water, the peaks for MM become less resolved, as expected from the well-known equilibria of HCHO that favor the formation of methylene glycol and, in turn, of larger polymeric species (Walker, 1964b; Moedritzer and Wazer, 1966; Dankelman and Daemen, 1976; Hahnenstein et al., 1994; Gaca et al., 2014; Gaca-Zajac et al., 2018). Therefore, it follows that formaldehyde, H_2_C=O, in CH_3_OH does not exist in the aldehyde form as the main chemical species. Instead, HCHO is locked in equilibria between the production of MM and the formation of hydrated species (Figure 7). This equilibrium with CH_3_OH is relevant for the analysis of HCHO in non-aqueous solvents to quantify the product of CH_3_OH oxidation when used as a benchmark for catalytic or photocatalytic activity. We demonstrate the ESI analysis of HCHO from a non-aqueous TiO_2_ suspension in methanol. To the best of our knowledge, this is the first report of the equilibrium between HCHO, neat CH_3_OH, and methoxymethanol and has implications for the analysis of formaldehyde because it enables extraction and preconcentration of HCHO using alcohols and non-aqueous solvents.

## Data Availability

The original contributions presented in the study are included in the article/Supplementary Material, further inquiries can be directed to the corresponding author.

## Footnotes

1 Subedi, P., Gutierrez-Portocarrero, S., Spain, S., Tucker, M. J., and Alpuche-Aviles, M. A. (2021). NMR and FTIR of Methoxymethanol. manuscript in preparation.
