# Supplementary figures and images for: Formaldehyde Analysis in Non-Aqueous Methanol Solutions by Infrared Spectroscopy and Electrospray Ionization
Source: Front Chem. 2021 Jul 2;9:678112. doi: 10.3389/fchem.2021.678112 (PMC8283199; doi:10.3389/fchem.2021.678112)

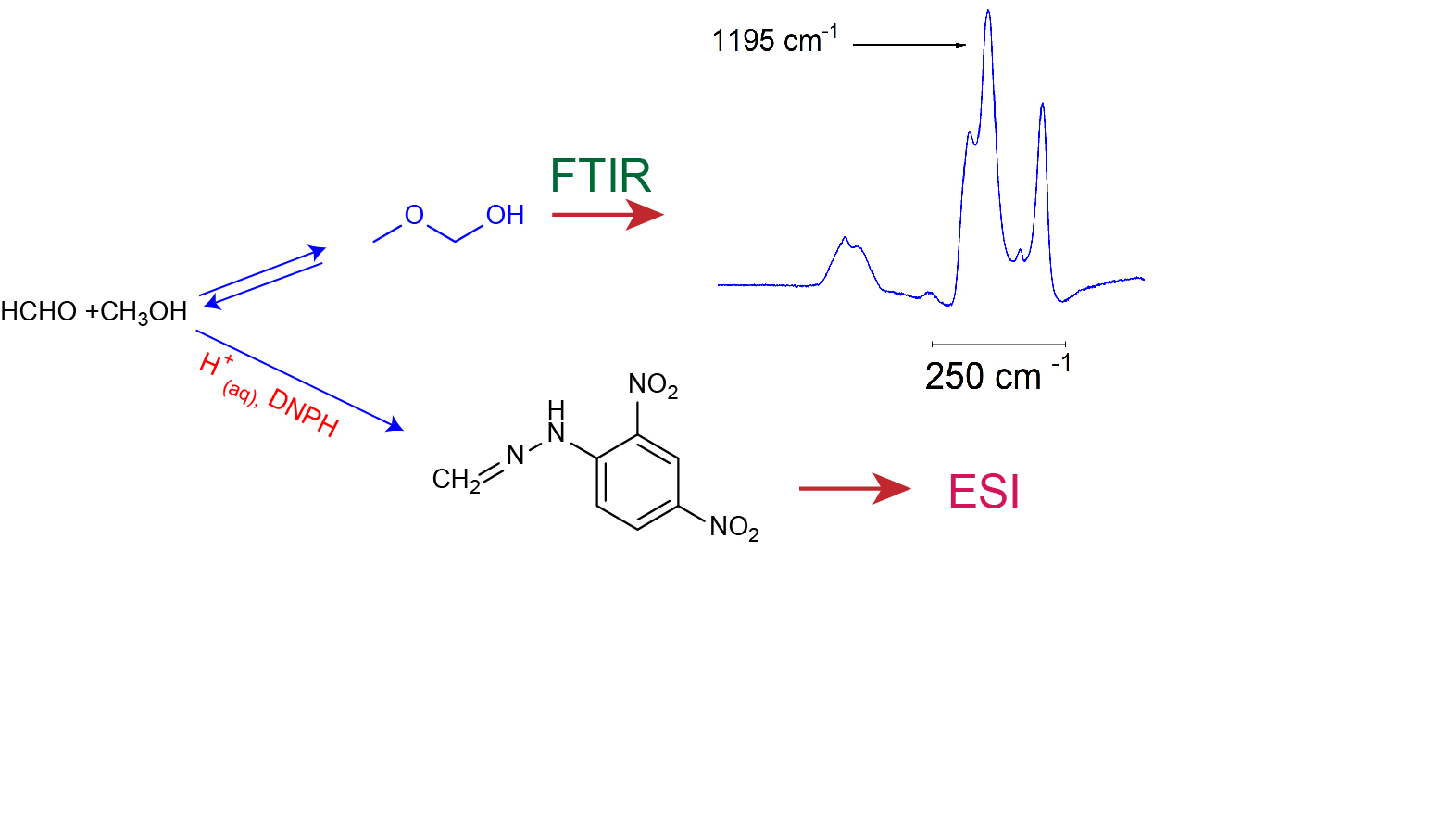

Supplement: Supplementary file 1 [file Image1.JPEG]
