# Supplementary material for: Formaldehyde Analysis in Non-Aqueous Methanol Solutions by Infrared Spectroscopy and Electrospray Ionization
Source: Front Chem. 2021 Jul 2;9:678112. doi: 10.3389/fchem.2021.678112 (PMC8283199; doi:10.3389/fchem.2021.678112)
Supplement: Supplementary file 2 [file DataSheet1.PDF]

Supplementary Materials

Formaldehyde analysis in nonaqueous methanol solutions by infrared spectroscopy and electrospray ionization.

Krishna K. Barakoti,[+] Pradeep Subedi,[+] Farzaneh Chalyavi, Salvador Gutierrez-Portocarrero, Matthew J. Tucker and Mario A. Alpuche-Aviles*

(+) These authors contributed equally to this manuscript.

Department of Chemistry, University of Nevada, Reno, Nevada 89557, USA

Table of Contents

I. Experimental Details 2

II. FTIR 3

III. Computational Vibration Modes 4

IV. Optimizing ESI MS of Formaldehyde 4

I. Experimental Details

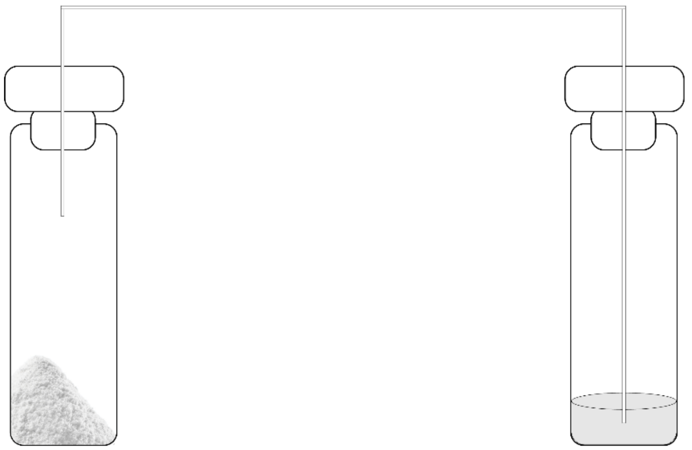

Figure S1. Schematics of apparatus used for paraformaldehyde cracking.

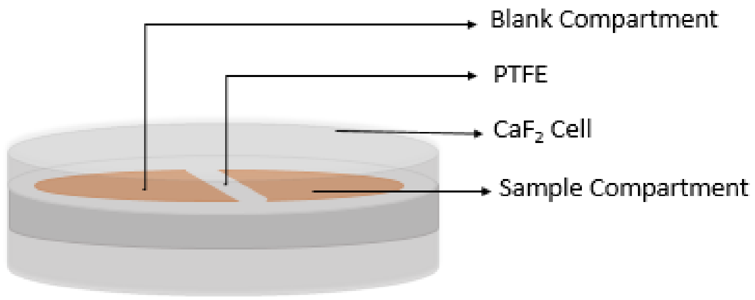

Figure S2. Schematic for FTIR cell setup with two CaF_2

II. FTIR

a)

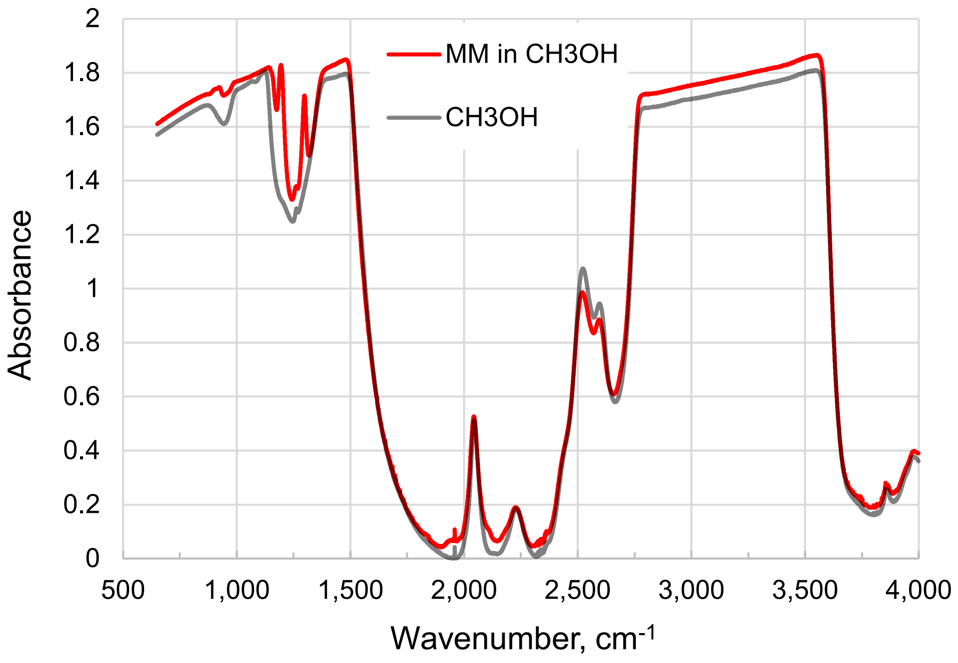

b)

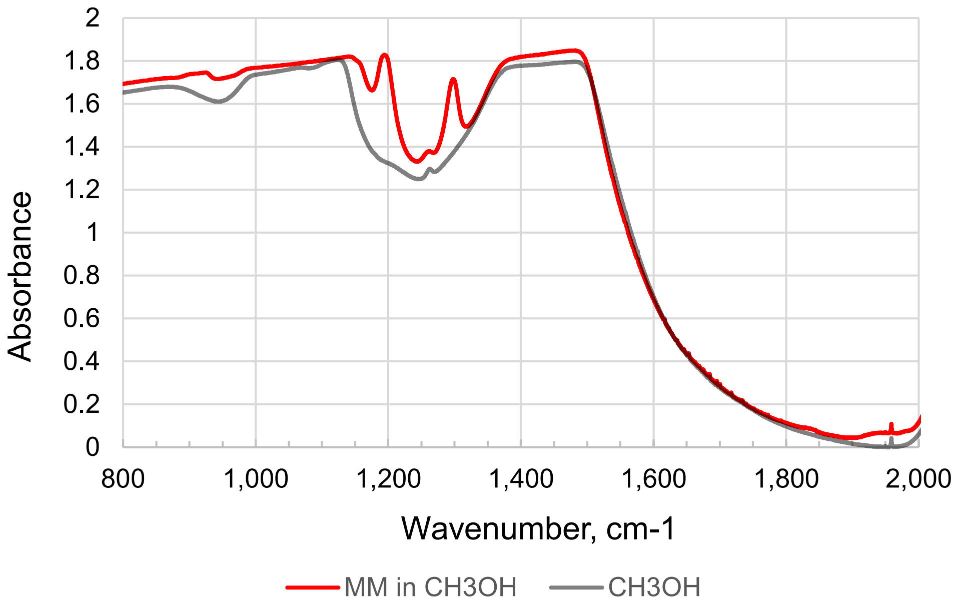

Figure S3. FTIR raw data for (a) methanol blank (———, black) and after purging cracked paraformaldehyde through the solvent to generate methoxymethanol (———, red). b) the region between 800 and 2,000 wave numbers. Note that the absence of a HCHO peak around 1,800 is not due to the detector saturating because A < 1 in this region.

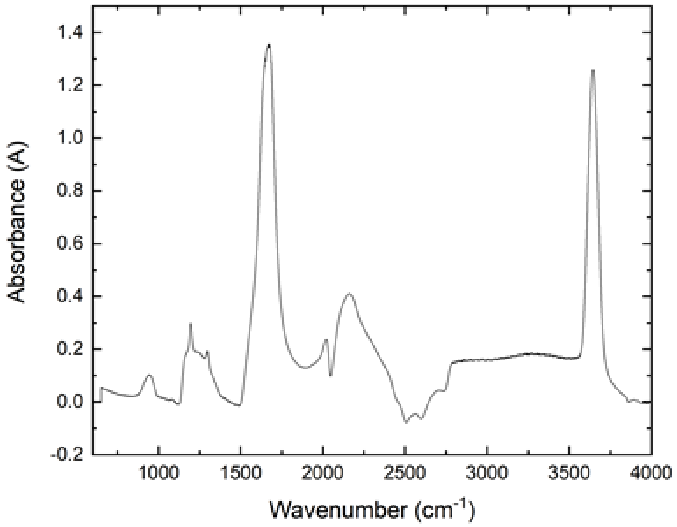

Figure S4. FTIR spectrum of a mixture of methoxymethanol and methylene glycol prepared by dissolving formaldehyde in anhydrous methanol and adding 150 μL deionized water.

III. Computational Vibration Modes

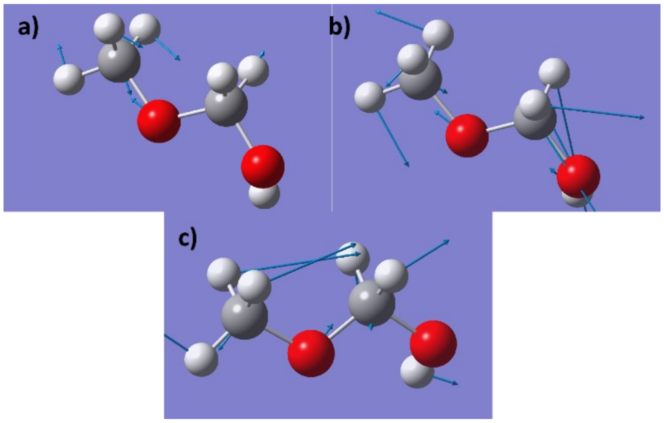

Figure S5 Calculated normal mode displacements for a) 1116 cm^{-1} b) 1195 cm^{-1} and c) 1297 cm^{-1} vibrational transitions determined for ab initio anharmonic frequency calculations. (C and O atoms are colored gray and red, respectively).

IV. Optimizing ESI MS of Formaldehyde

Sulfate Effect: Our initial attempt to quantify HCHO without removing sulfate resulted in anhydrous MeOH resulted in a calibration curve with lower intensity and a relatively low R^2 of 0.94 (**Figure S6**)

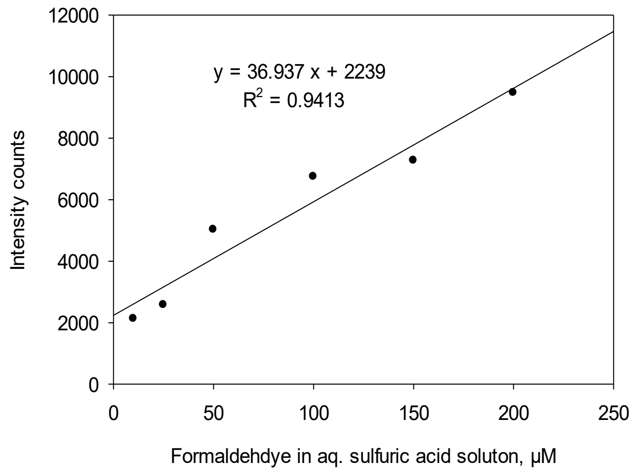

Figure S6. Calibration curve for standard formaldehyde from aqueous standard solution, obtained by derivatization without an extraction protocol. Note signal intensity count shown in the curve is for mass fragment, m/z =209.01.

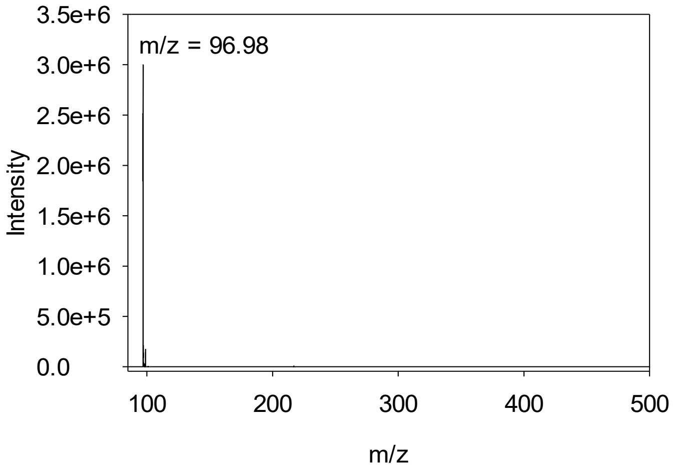

Figure S7. ESI spectrum assigned to HSO_4^- for derivatized formaldehyde solution prepared from 150 µM formaldehyde in methanol solution with 2, 4 DNPH in 0.5 M sulfuric acid.

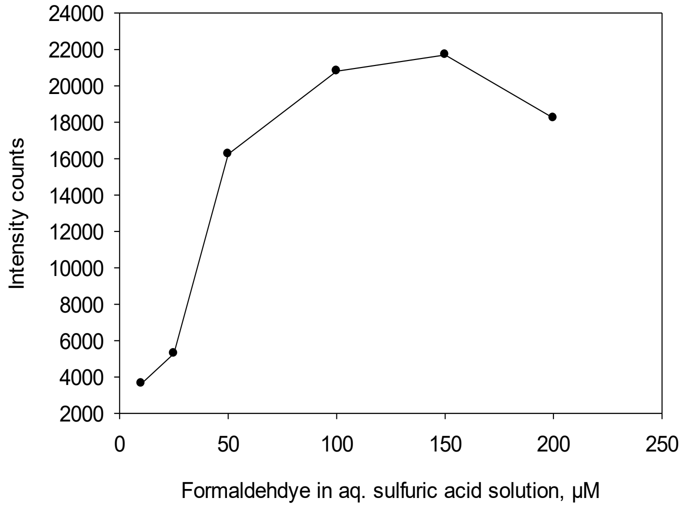

Figure S8. Calibration curve obtained for formaldehyde derivative (m/z =209.01) after treatment with ammonium hydroxide. Note: formaldehyde in aq. 0.5 M sulfuric acid is the starting solution.

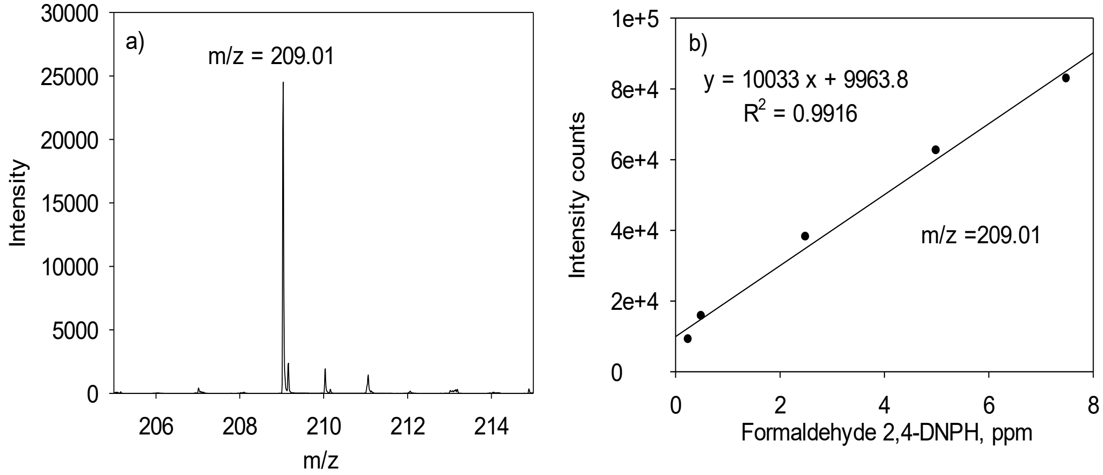

Figure S9. ESI spectrum and calibration curve obtained for formaldehyde 2, 4-dinitrophenyl hydrazone (standard) prepared in methanol and isobutyl acetate mixture a) spectrum for formaldehyde 2, 4-Dinitrophenyl hydrazone (standard) b) calibration curve for formaldehyde 2, 4-Dinitropheyl hydrazone (standard) prepared in 1:1 v/v methanol and isobutyl acetate mixture
